# The Hospital Nursing Supplement

**Published:** 1894-11-10

**Authors:** 


"The Hospital" Nursing Mirror.

Being the Extra Nursing Supplement of "The Hospital" Newspaper.

[Contributions for this Supplement should be addressed to the Editor, The Hospital, 428, Strand, London, W.C., and should have the word "Nursing" plainly written in left-hand top corner of the envelope.]

### News from the Nursing World.

### OUR PRINCESS.

The long journey lasting from half-past eight a.m. on Wednesday, when their Royal Highnesses the Prince and Princess of Wales left London, until Sunday morning when they reached Livadia, has aroused much interest in our nurses. The love and respect felt for "Our Princess," the President of the Royal National Pension Fund, make all news of her welcome, and it is with heartfelt sympathy that the thoughts of our readers have followed her on this weary journey, and in the sad interviews which awaited her in beautiful Livadia.

### THE QUEEN OF GREECE.

The knowledge of nursing acquired by the Queen of Greece in the hospital at Athens and elsewhere rendered her presence peculiarly comforting and helpful to the Czarina during the recent sad events in the Russian Royal family.

### WANDERING NURSES.

The address of the new offices of the Royal National Pension Fund for Nurses is 28, Finsbury Pavement, and a more central spot could not easily be found. The number should be carefully noted by all policy-holders for their own personal convenience, as complaints reach us that owing to the insertion of "20" instead of "28" in a contemporary, some of our readers have wasted a little of their own and other people's time and patience.

### AMBULANCE WORK.

Railway accidents, like epidemics, generally occur when least expected, and therefore, as has been recently pointed out, it is far more useful for all classes to be instructed in giving "first aid" than it is to establish an elaborate "equipment" at great centres. Knowledge, like "appliances," needing to be widely spread to be of general use. Much encouragement is given to miners and their wives, as well as to railway servants, in qualifying to act promptly in emergencies, and this is one of the best outcomes of the St. John Ambulance Association. The directors of the North-Eastern Railway set an excellent example in securing instruction for their employés. Last Saturday a capital display took place at Stratford, when five ambulance teams from different districts had their final competition for a challenge cup. A large number of spectators assembled in the Great Eastern Railway Company's wagon works, and an address was afterwards given by Lord Claude Hamilton. The men showed remarkable dexterity, and their proceedings aroused great interest in the on-lookers.

### MALE NURSES IN THE ARMY.

The recruiting and training of men for the Army Medical Staff Corps are subjects under discussion at present, the *British Medical Journal* pertinently suggesting that the depôt should again be stationed at Netley. This would ensure ward training and experience in nursing for the orderlies at the Royal Victoria Hospital.

### REPRESENTATIVE OR REGISTERED?

Any question as to whether the Matrons' Council aspires to be a representative or a party association appears to have been set at rest at last week's meetings. The suggestion of one member as to the narrowing influence which might be exercised by the insertion of the word "Registration" in the bye-laws seems to have been quenched by an assertion that many important and influential hospitals were ripe for it. Although the names of these institutions were not given, the expression "state registration" obtained some favour in spite of attention being called to the fact that it did not exist. The decision as to whether the executive or the council should be responsible for the admission of new members and associates is said to have been postponed in order that legal advice might be taken as to the amount of protection which could be claimed by ladies on whom, perchance, might fall the ungracious task of declining the application of a professional sister. At present, therefore, the Matrons' Council is composed of associates who are not entitled to all the privileges of membership, and of members who are or have been matrons or superintendents. Nothing prevents a woman who has for a short period presided over even a cottage hospital from retaining a seat on the council after her return to the subordinate position of ward sister. The position held by a body thus constituted can easily be foreseen, and we leave our readers to form their own opinions on the report which appears in another column.

### WHERE WAS THE DOCTOR?

A most pathetic illustration of inadequate nursing is reported from Cardiff, where an unfortunate man died in the union workhouse from the effects of arsenic poisoning, and the night nurse "could not give him much attention as she had ninety-four other patients to attend." The police-constable, according to the local press, swore that the patient appeared to be in pain all the night, and by midnight had got considerably worse, and this statement he repeated before the coroner. Yet the nurse is reported to have considered the patient "all right" at 4 a.m., and when he asked her to stay with him she told him it was impossible. At 4.55 the police-constable announced the man's death, and most people will agree with the "rider" to the verdict at the inquest, that "the jury desire to express their surprise that so large a number of sick patients should be entrusted to the charge of one nurse at the Cardiff Union Workhouse." The poor man, whose remorse for his suicidal act evidently added mental to his bodily sufferings, was apparently altogether without such skilled care and kindness as are the due of the dying. The Coroner, in summing up, said that "it was not pleasant" to think that the poor man "should have received no treatment during all those hours."

### ADDENBROOKE'S HOSPITAL, CAMBRIDGE.

There is a vigour and energy about the committee of this institution when it is once moved which is most refreshing. They have already determined to recommend to the next court of governors that the three years' system of training for probationers shall be introduced forthwith. This is only one of several important changes which we have every confidence will now be made at Cambridge without delay or hesitation.

### "BORN TO MEASLES."

The recent discussion regarding an account due for the services of extra nurses employed at the Kidderminster Workhouse is chiefly interesting for the views expressed by the Guardians. The Rev. W. Finch asserted that ordinary persons can effectively nurse typhoid fever, and that "people were born to measles, and measles were as common as blackberries, and did not require a special nurse." If the local press report is accurate, we must believe that some members of the Kidderminster Board hold very obsolete ideas as to modern treatment of disease. The terrible mortality amongst children who contract measles, and are not well nursed, shows the urgent need for skilled care being given to patients of a class in which poverty is frequently conjoined with ignorance. The medical officer at the workhouse considered efficient nursing economical, and spoke of the proper and humane way in which the doctor's orders were carried out by skilled nurses. It is evident that alterations are required in the nursing arrangements at Kidderminster, an adequate staff of resident nurses being essential.

### STALEYBRIDGE.

The thirteenth annual report of the Staleybridge Sick Nursing and Mothers' Help Association gives an excellent account of the work achieved during the past year. The committee speak with special pleasure of the nurses having served them faithfully and remaining with them. Nurse Twamley was trained at Crumpsall Infirmary, Manchester; Nurse Farrand at St. Mary's Hospital, in the same town; and Night Nurse Bailey received her training at the Ashton-under-Lyne Infirmary. A variety of medical appliances are sent out to patients at specified charges, and invalid nourishment is furnished to acute cases when necessary.

### PARIS.

The management of the Hospice des Enfants Assistés at Paris has been vastly improved, and the attention and treatment of the children during convalescence receive special care. The results are most satisfactory, and the elaborate precautions exercised for isolation and disinfection have materially diminished the infant mortality from infectious diseases. The Hospice must be a valuable training school for children's nurses.

### DANGER SIGNALS.

The use of placards to be placed on the doors of apartments where infectious diseases are being nursed has been sanctioned by the New York Board of Health. Diphtheria is to be labelled in white, scarlet fever in red, and measles in blue. The placarding of houses in Ceylon is no novelty, and it will be interesting to learn if the system is found more efficacious in stamping out disease in New York than it has elsewhere proved.

### TEACHERS AND TAUGHT.

"Don't you find rural audiences very sleepy?" asked a would-be health lecturer. "Only when I am dull," was the brisk response of the experienced teacher. Surely this reply embodies a useful lesson. Instruction must be suited to the pupils, the latter cannot be selected to suit the teacher. Whether in district or private work or as a lecturer in rural districts a woman must learn to understand and sympathise with those she addresses. If they are unwilling to follow her guidance, she may rest assured that she has failed to make her teaching or services acceptable.

### PRIVATE NURSES IN SOUTH WALES.

An association of private nurses has been established at Cardiff by Miss Annie Mackie, 152, Newport Road. Nurses are admitted to the general staff after two years' hospital training, and they receive their own fees less 10 per cent. on their earnings. The charges for board and residence at the Home between cases appear moderate. Miss Mackie thinks she shall be able to reduce the percentage paid by the nurses when she has a larger staff at the institution, which she has named the South Wales Nurses' Co-operation.

### THE PROFITS OF PRIVATE NURSES.

In Bombay, according to the *Pioneer Mail*, the nursing at the European General Hospital is mainly supported out of the profits made on "outside" cases. In other words, the nurse who takes private patients is (perhaps unwittingly) the chief contributor to the maintenance of the hospital nursing staff.

### FRIEDENHEIM.

An anniversary meeting on behalf of this "Home of Peace" was held in the Portman Rooms on November 7th. The Rev. Prebendary Webb-Peploe was in the chair, and speeches were made on behalf of the institution by Mr. Hugh Mathieson, Rev. Henry Sharpe, Mr. John Langton, F.R.C.S., and others. All spoke with much appreciation of the work which had been organised and carried on by Miss Davidson, and of the beautiful Home into which the new premises have been converted. Friedenheim (of which their Royal Highnesses the Duchess of Teck and the Duchess of York are patrons) is situated near the Swiss Cottage. The Home can now accommodate forty patients, and 112 were treated in the course of last year; of these sixty-eight died, twenty-one have been discharged, and twenty-three remain under treatment.

### SHORT ITEMS.

The Medical Aid Society for Gentlewomen in reduced circumstances has issued its fourteenth annual report, and appeals urgently for new subscribers.—The "Nursing Talks" announced in "Where to Go" this week are specially suitable for young mothers and others interested in the care of children. —The Pudsey branch of the British Women's Temperance Association have decided to set up a nurse for the sick poor of the district; the working men in the neighbourhood have willingly subscribed to the fund.—Miss L. Kirkley, who for several years has been a nurse at Sherburn Hospital, was recently married to Mr. James Glenn, head gardener at Retford Rectory. Nurse Kirkley received many gifts from friends and fellow workers.—The Duchess of Bedford has given £10 to the Workhouse Infirmary Nursing Association, and has become a member of the general committee. —The directors of the tramway company at Dundee allow the district nurses to travel at reduced fares.—The Duchess of Teck visited the Chelsea Hospital for Women on the 5th inst. in company with Prince George of Teck, and gave away flowers to the patients.

### Notes from St. Helena.

By Rose A. Blennerhassett, Lady Superintendent of the Civil Hospital.

### I.—ARRIVAL AT ST. HELENA.

Day was just dawning on the morning of December 19th, 1893, when the U.S.S. "Gaul" anchored off St. Helena. We peeped out of our porthole and caught a glimpse of a looming mass of rock, apparently cleft in twain at one spot, a church spire, a fleet of little boats putting off from the shore and making for the "Gaul." Reflecting that we should have ample opportunities of becoming thoroughly acquainted with the island in all its aspects, we went back to bed and fell asleep, only to be roused by the stewardess, who informed us that the colonial surgeon was actually on board at that unearthly hour, and wished to make our acquaintance. We sent a polite message to explain that this was impossible for the moment. As straws show the direction of the wind, so did this little incident reveal the absolute emptiness of life at St. Helena. Hardly had we finished dressing when a message reached us from the Governor requesting us to go to the Castle as soon as we landed.

### Homeless.

Through the messenger we learnt that the fate which pursued us in Africa had overtaken us here. We were homeless; our quarters could not possibly be ready for us in less than a month or six weeks. Of course, our first impulse was to rush to the captain and say, "Take us away—to the ends of the earth—land us anywhere rather than here." But copy-books warn us to beware of those first impulses (which are almost always good), so we held our peace, went to breakfast, and after that repast had ourselves put on shore.

As we landed we met the colonial surgeon, a young fellow, in appearance very like Rudyard Kipling, who greeted us cordially, and begged us to make use of his carriage. This was a quaint hybrid vehicle, neither bath-chair nor Victoria, but reminding us of both. These little carriages are in common use here, and are harnessed to a single pony, which a boy leads up and down the steep hills. Imagine a chart which records a temperature that races up and down between 97 and 104 degrees, and the result will give a very good idea of St. Helena roads.

### Interview with the Governor.

The Castle of St. Helena, a large solid house with few architectural pretentions, faces the harbour, and was soon reached. Alighting in the courtyard, we were shown into the Governor's office. His Excellency received us with much kindness, and asked us to go to "Plantation," as Government House is called here, until after Christmas, as the hospital was not to be taken over until January 1st. We felt that this was most kind and thoughtful, for few people care to invite utter strangers to spend Christmas with them. It was settled then that we should drive up to Plantation that afternoon and meanwhile inspect the Civil Hospital. The Governor told us that it was in a deplorable state, but even this description fell short of the reality.

### Jamestown.

Reseating ourselves in the doctor's carriage we were led up a long tortuous street, sloping uphill away from the sea. The houses on each side were for the most part small and irregular—some of them mere cottages, others large, showing traces of having once been well inhabited and carefully kept. Jamestown is built in a narrow ravine shut in closely by walls of rock, barren save for growths of prickly pear. These hills so overshadow the town that the rocks not infrequently roll down into the streets, wrecking houses, and sometimes killing the inhabitants.

### The Civil Hospital.

The hospital stands at the end of the town farthest from the sea. On either side it looks upon walls of rock, the effect being exceedingly gloomy. The building stands back from the street, in a small enclosure in which a few banana canes and oleander bushes grow. As we drove up a troop of noisy street boys, who were playing in the enclosure, rushed out whooping and dispersed in all directions. An air of neglect and decay pervaded the place.

### Nurses at Rio de Janeiro.

### YELLOW FEVER AND A PRESENTATION.

There stands a magnificent yellow fever hospital on the opposite side of the harbour at Jura Juba, a charming place, beautifully fitted up, the tiled rooms looking so cool and clean. There we saw a different style of treatment; no washing is done to the patients, not even a bath until they recover. We saw some men from coaling ships in bed covered with coal dust, but the attendant told us it was not considered a good thing to wash them; we had heard of this before, but thought it was perhaps an exaggeration, but no, it is perfectly true. We went and chatted to those who spoke English; they seemed very pleased to see us, and all spoke highly of the kindness they received.

We must not omit to tell you of the presentation from the ship captains to the nurses of the Strangers' Hospital.

We have already mentioned how grateful they were for the care received. After they left they met in conclave, and decided that their appreciation should take a substantial form. Of course, when the nursing staff heard this they requested that whatever gift was intended should go to the hospital. This was refused, and after consultation they decided upon gold crosses. They were made here—very handsome crosses on chains, with the recipient's name and reason of the gift inscribed thereon. They were presented by some of the senior captains then in the harbour. In spite of the danger, the satisfaction of being of use to sufferers in a strange land is a reward in itself, independently of the deep gratitude expressed by patients in more ways than one. And after all, diphtheria, small-pox, and typhus are dangers in our own native land. We are not safe there; indeed, where have we not something to brave?

We have omitted from time to time to mention that there is no opening for private nurses at present. During the two years we have been established only ten applications have been made for them, and some of the cases have been very short. Hotel expenses are so great, it would be impossible to get a bare livelihood, except in the yellow fever season.

There has just been another grand ball in aid of the Strangers' Hospital, by which it is hoped a fair sum will be realised.

Cornstalk.

### Death in our Ranks.

We hear from Pietermaritzburg of the death of Nurse E. Barbara Harrison at the Nurses' Institute in that town. She was trained at St. Marylebone Infirmary, and joined the Royal National Pension Fund in 1890. Last January her health broke down and she received sick pay for nineteen weeks. After undergoing operations for enlarged glands, a sea voyage was recommended, and in July Miss Harrison left England. Her last illness was a very short and unexpected one, and she was nursed by a former fellow nurse who had preceded her to Natal from St. Marylebone Infirmary. Miss Harrison was much beloved, and is greatly regretted by many friends.

### Appointments.

Cambridge Infectious Diseases Hospital.—Miss Norris has been appointed Matron of this hospital, where she has held the post of Nurse. We congratulate her on her promotion.

Leyton Children's and General Hospital for Walthamstow.—Miss Clarissa Hunter has been made Matron of this hospital. She was trained at St. Bartholomew's Hospital, and worked there for seven and a-half years, and at the Foundling Hospital for two years. We wish her all success in her new work.

### News from Victoria.

By Our Own Correspondent.

The months of July and August in Victoria are the time for "annual meetings" of charitable institutions of all kinds—hospitals, asylums, orphanages, homes, societies—as these partake of the Government funds, and their year ends with the Government financial year, June 30th. Each and all of them have been sending up the same plaint—decreased funds and more to do with them. The most important of these institutions, the Melbourne Hospital, is £14,000 in debt. This, of course, is the accumulated deficit of several years, and against it must be set the fact that the institution has an endowment fund, but naturally there is a disinclination to trench upon the endowment. At the annual meeting of the governors of the Melbourne Hospital, held at the institution on July 25th, the president, Mr. F. R. Godfrey, who occupied the chair, made a strong appeal for the exemption of the hospital from reduction in the amount of the Government grant allotted to it. A further reduction of £2,000 on the last year is chargeable from the fact that the committee have been unable to keep their outgoing within their income. The grounds of exemption urged are that the Melbourne Hospital takes in any and every case that applies, cases that other institutions cannot or will not admit. During the year just ended, 362 cases of diphtheria alone were received, 464 patients were treated in the refractory wards; and of the 600 deaths, 136 took place within 72 hours of admission. It was pointed out that private subscriptions had fallen off in comparison with the "boom" years only, not with normal years, so that the reduction of the grant in aid is mainly chargeable with the deficit.

The Women's Hospital has treated 1,406 in-patients and 1,642 out-patients, the largest number it has ever had, and any deficiency in its accounts has been prevented by a number of successful "special appeals" and a contribution of £500 from the estate of the late Mr. T. J. Sumner. A pressing necessity at present is the enlargement of the gynæcological wards, a large and ever-increasing number of cases awaiting operations, for whom there is no room. The committee are reluctant in the present juncture to commit themselves to further expenditure, but the need is so great that it will probably be forced upon them.

The merits and the needs of the Austin Hospital for Incurables are now, fortunately, becoming better known and appreciated, and a large ball in aid of the funds, which was given in June, realized over £300, and a number of smaller entertainments and others still in prospect are for the same object. As the expenditure of this institution is £11 per day, and its ordinary income from all sources is not £6 per day, there is evidently room for much charitable effort to keep it afloat. There is, indeed, a strong feeling that the whole charitable system of the Colony must be put on a new footing The governors of the Melbourne Hospital advocate a special rate. A Bill dealing with the matter has been prepared, and should have been presented to Parliament early this session, but has been shelved during the Budget debates, and with a dissolution imminent will probably have to wait another year.

The annual meeting of the Charity Organisation Society on July 16th was not allowed to pass without a reference by Professor Morris to his letter to the *Times*, which was so unanimously pronounced ill-judged and ill-timed. Professor Morris held to his opinion that the letter—left alone—would have done some good and not harm, by securing money from absentees. He said he had had a letter from a friend who was in the Reform Club when a wealthy Australian said he had, on seeing Professor Morris's letter, intended to send a cheque, but that seeing another letter a day or two after, he had refrained. Dubious laughter greeted this story, it being a fact that our "absentee landlords" do little in the way of maintaining charity.

Mr. Topp, Chairman of the Central Board of Health, having been promoted in July, the Government following out their retrenchment policy indicated by a Bill a desire that Dr. D. A. Gresswell, medical expert to the board, should also officiate as chairman and do the work of the two positions for one salary. The members of the board have the right to elect their chairman, and several very lively discussions took plate, most of the members being of the opinion that it was a pity to hamper Dr. Gresswell, who stands alone here in the extent of his sanitary knowledge, with other duties. As there seemed to be no one else, however, particularly adapted for chairman, the matter ended in the election of Dr. Gresswell.

A conference, convened by the Central Board of Health upon the requirements of the metropolis for effectually dealing with first cases of small-pox took place in the middle of July. It was attended by about 35 delegates and most of the metropolitan municipalities were represented. These were practically unanimous in rejecting the view that the municipalities should bear any share of expense in such matters. This means that Melbourne is to be left some considerable time longer unprotected from this fell disease, which might at any time become endemic, according to Dr. Gresswell's opinion.

Victoria as yet does not possess a crematorium, but this defect is likely to be remedied shortly. At a meeting of the trustees of the St. Kilda Cemetery Trust on August 7th, the resolution proposed by Mr. F. R. Godfrey, that: "It is desirable that a crematorium be established in the St. Kilda Cemetery," was voted unanimously. Mr. Henry Gyles Turner, Chairman of the Trust and Inspector-General of the Commercial Bank, said: "If it could be established legally, without objection to neighbours, and providing the Trust had the money, he thought it advisable to make the experiment." Mr. H. K. Rusden, Parliamentary Librarian and secretary of the Cremation Society of Victoria, wrote announcing his intention of putting a clause in his will directing that his body should be cremated at St. Kilda if such a building were erected. St. Kilda is a seaside suburb of Melbourne, and the cemetery, which is nearly full, is surrounded by houses. Mr. F. R. Godfrey recently returned from Europe, where he examined the question of cremation *versus* burial.

The various provisions of our Health Act have hitherto been extremely inaccessible, to be found only by laborious research through old files of the *Government Gazette*; but Mr. H. S. Cole, LL.B., barrister and solicitor, and Mr. Thomas Morris have just issued a manual on "The Health Act, the Infant Life Protection Act, and the Margarine Act," wherein these are to be found in handy form, with the valuable addition of a full and complete index. In this work these Acts are simplified for the benefit of the ordinary lay understanding, cross references being given where necessary to widely-separated but cognate sections. The Victorian Health Act is dated 1890, and deals with a vast variety of matters—quarantine, the cleansing of streets, hospitals, pig keeping—in which it probably resembles the English one. The new manual is a boon to laymen.

A new Australian medical journal, *The Inter-Colonial Quarterly Journal of Medicine and Surgery*, has reached its second number.

### Wants and Workers.

Can anyone tell me whether English nurses are employed in Freemantle or Perth in Western Australia?—Nurse A. E. W.

### The Glasgow System for Probationers.

The first sessional lecture in connection with the Matron's Council was held at the Medical Society's rooms, 11, Chandos Street, the same evening. Miss Isla Stewart was in the chair, and there were present about forty nurses with a small proportion of matrons and medical men. Mrs. Strong, Matron of the Royal Infirmary, Glasgow, read an interesting paper, entitled, "The Method of Training Nurses in the Royal Infirmary, Glasgow, and a Plea for Uniformity in the Education of Nurses." In the course of her remarks the lecturer laid stress on the desirability of preliminary teaching and consequent examination of all probationers before admission to the practical work of a hospital ward. At Glasgow every candidate has to undergo a three months' course of theoretical work before entering the infirmary. She attends classes in Anatomy, Physiology, and Hygiene, and devotes her whole time to these subjects. On completion of the term an examination is held, and if found competent, the candidate is admitted to the "Home." Her practical work in the wards then begins, eighteen months being spent in the female and eighteen in the male wards. In advocating the system Mrs. Strong laid special stress on the advantages obtained by supplementing the theoretical with the practical work, instead of carrying on both at the same time. Having mastered the theory, a probationer can follow with greater intelligence the clinical lectures delivered to the students, and also understand the reason why she has to carry out the instructions given her. The expenses of training have to be defrayed by the probationer in the form of a fee.

The necessity for a uniform system of education, a central board of examiners, and recognition by the State of registration of nurses were urged; also the legal definition of the curriculum through which a nurse should pass and the standard to be attained before she be considered "trained." A graceful allusion was made in conclusion to the painstaking and devoted efforts made by H.R.H. Princess Christian to raise the status of professional nursing in the United Kingdom. A discussion then ensued, in which Miss Isla Stewart, Mrs. Bedford Fenwick, Miss Kenealy, Dr. Sydney Coupland, Miss Mollett, and others took part. Dr. Coupland considered that if fees were enforced a good many excellent women would be excluded from the profession on account of their inability to pay them. Miss Mollett argued that, with regard to the preliminary scientific course, many of its advantages would fail when applied to the smaller hospitals without medical schools, and on these such a regulation would press hardly. Mrs. Spencer asked whether the preliminary examination ought not to include also one in practical work, which was most important in a nurse, as many women had admirable theoretical knowledge, but no practical fitness whatever for the mere domestic duties of their vocation. On conclusion of the debate, the following resolution was put to the meeting by Miss Isla Stewart: "That this meeting is of opinion that an Act of Parliament should be obtained to provide for the uniform training, the uniform qualification, and legal registration of nurses." This was seconded by Miss Kenealy, and carried. A vote of thanks to Mrs. Strong for her paper and to Miss Stewart for presiding concluded the proceedings, after which the company partook of coffee and light refreshments.

### Presentations.

The friends and patients of Nurse McArdie presented her with a case of surgical instruments, bearing a suitable inscription, on her retirement from Burslem, Staffordshire, where her services have been highly valued by the sick to whom she has ministered for over two years.

A handsome salver was presented to Miss Allen on October 30th by the nursing staff of the General Hospital, Birmingham, on the occasion of her giving up the post of Matron which she has so ably filled. Many regrets were expressed at Miss Allen's approaching departure, and an excellent farewell concert was given in one of the wards, which was largely attended.

### The Matrons' Council.

The first annual meeting of the Matrons' Council was held on November 1st, at the Matrons' House, St. Bartholomew's Hospital, Miss Isla Stewart taking the chair. Rather over a score of members were present, and the proceedings were conducted in a fairly business-like manner. Attention was chiefly given to the drafted bye-laws, most of which were discussed at some length. As they appeared in The Hospital of July 21st, our readers are doubtless familiar with their purport. Two alterations have now been made in the paragraphs relating to the objects of the society, in (*a*) and (*c*) the words "hospital matrons" being replaced by the words "the members;" in (*b*) there is an addition of "state registration" to follow the words "uniform system of education, examination, and certification." The suggestion that it was desirable to make a distinction between "women who are" and "women who have been" matrons and superintendents was not favourably received, and was withdrawn. A new bye-law was carried, to rank as No. 4. "Associates may attend conferences at which they may read papers and debate; they are not eligible for official positions, or to sit in council or on the Executive Committee. The Hon. Secretary may be an associate not engaged in active duty." Other unimportant alterations were duly proposed and seconded. After an exhaustive discussion on rule 5 (which will in future stand as 6), decision on it was postponed that legal advice might be obtained. Thus it remains uncertain whether the Council or the Executive Committee will be responsible for the acceptance or rejection of candidates for associateship or membership. There are to be three instead of two vice-chairmen, as at first proposed, one to retire each year in rotation, and of the twenty members of the Executive Committee a quarter will also retire annually in rotation.

### Where to Go.

Trained Nurses' Club, 12, Buckingham Street, Strand.—On Tuesdays, at three o'clock, "Nursing Talks" will be given at this club; fee for course, 7s. Subjects: November 13th, "Hygiene"; November 20th, "Diet and Medicine for Children"; November 27th, "Sick Children"; December 4th, "Infectious Diseases of Children"; December 11th, "Cooking in the Nursery"; December 18th, "Nursery Management." Tickets obtained from secretary, or from Miss Robinson, 95, Philbeach Gardens, S.W.

Miss De Pledge, Matron of Chelsea Infirmary, will give six lectures on Sick Nursing at 53, Berners Street, on November 9th, 16th, 23rd, 30th, and December 7th and 14th, at half-past two p m.

Midwives' Institute, 12, Buckingham Street, Strand.—Mrs. Scharleib, M.D., B.S., will lecture on "Puerperal Septicæmia" at a quarter to eight on November 16th. Non-members are admitted at 6d. each.

Drawing-room sale of work at 93, Abingdon Road, Kensington, on November 23rd, for the Zenana Bible and Medical Mission.

It is announced that two dances will be held this season—the dates are Thursdays—December 13th and January 10th—in aid of the Chelsea Hospital for Women, at the Princes' Hall.

### Notes and Queries.

### Queries.

(32) *S. Africa.*—Information is sought as to the prospects of nurses in Africa.—*Nurse Dorothy.*

(33) *Name.*—I am desirous of verifying the name of a doctor which I saw in a medical paper last year. Could one of your readers help me?—*Brixham.*

(34) *Hospitals.*—Where can I get information as to cottage and other hospitals?—*A. W.*

(35) *Australia.*—Is there a good opening for nurses in Western Australia?—*A. E.*

### Answers.

(32) *S. Africa* (*Nurse Dorothy*).—You will find information on this subject by looking back in The Hospital, but nurses are not advised to go out unless they have friends to live with between cases or have private means.

(33) *Name* (*Brixham*).—You will find the information you desire in the Medical Directory.

(34) *Hospitals* (*A. W.*)—Get "Burdett's Hospital Annual," published by Scientific Press, 428, Strand, London.

(35) *Australia* (*A. E.*)—We are constantly told that the supply of nurses in the Colonies exceeds the demand. Doubtless many so-called nurses are quite incompetent for the work they profess to do, but you would be unwise to go out "on your own account." Colonial nurses are somewhat jealous of English-trained ones.

### Dress and Uniforms.

By a Matron and Superintendent of Nurses.

### The New Corselet.

Those who are anxious to combine health and comfort with the possession of an elegant and natural figure cannot do better than procure the Khiva Corselet. This corselet is constructed on entirely hygienic principles, and adapts itself with perfect ease to every movement of the body. The "Khiva" differs from all other forms of corset in that it is confined to the upper part of the body only. It ends at the waist in a series of tabs, to which the skirts are fastened by buttons. Nothing more comfortable for invalids, married ladies, or nurses can well be imagined, while the freedom from all pressure which is thus ensured renders it peculiarly adapted for all young people engaged in active exercise, such as tennis, boating, golf, or riding. The mechanism of this corselet is very simple, it is adjusted at the back with a buckle, and fastened in front with a wide hook at the top, and an elastic band at the waist, which is let into the corselet from the sides. Shoulder straps are another feature to be noted, as by these means the weight of the clothing is taken off the lower part of the body. These corselets can be had in all colours and prices from 5s. 11d. to 19s. 11d. They are beautifully finished off, very soft and pliable, and are guaranteed to wear out four pairs of ordinary corsets. All that will be found necessary in fitting is to take the measurements round the bust under the arms. Instructions for fixing are sent with every pair. Head Depôt, "Khiva" (Limited), 42, Poultry, London, E.

### Nursing Uniforms.

We should also recommend nurses who are in want of a uniform to pay a visit to the establishment of Messrs. Roberts at Islington, where many delightful novelties in this line are now to be seen. A neat little "Princess" bonnet, trimmed with black velvet, costing 6s. 11d., especially takes our fancy, and we believe will be most popular with those of our readers who are tempted to make an investment. Caps there are in every variety of shape. The "Marie" is not only becoming but very easily adjusted, and only requires the string untying to be ready for washing. The price with strings is 1s. 6d., but as it looks equally well without them they can be discarded, thus reducing the cost of the cap to 10¾d. Messrs. Roberts are selling a very nice apron, full size, for 1s. 7½d. It can also be had in a superior quality at a small increase of cost. In conclusion, we feel sure that no one will regret an hour or two spent in this enterprising establishment.

### Christmas Novelties.

The catalogue just issued by Mr. Robert French is one deserving special attention. Among the many charming articles therein depicted it is difficult to say which is most to be desired. Watches, jewellery, and an infinite variety of plated goods all claim our consideration in turn. For Christmas presents or presentation purposes such a selection will be found invaluable and save much trouble and anxiety to the intending purchaser. Very tempting is a beautiful little semi-hunter lady's watch, which can be had in silver for £1 10s., or in eighteen carat gold for £4. To a nurse such a present would be most acceptable if she does not happen already to possess one. A richly-mounted oak inkstand only costs 18s., and a very nice looking Tantalus cannot be called dear at £1 15s. Oak trays and biscuit boxes with plated mounts, and afternoon tea services are as moderate in price as they are fascinating in design. The fancy goods and hardware departments are equally attractive, but as our space is limited we would again refer our readers to the catalogue.

### ANSWERS TO CORRESPONDENTS.

Cuffs.—*Trained Nurse* would be glad to hear where she can obtain the cuffs to button to the sleeve, spoken of by "A Matron and Superintendent" in The Hospital Supplement of October 6th.—These cuffs can be procured from Messrs. Garrould, 150, Edgware Road, at 6¾d. per pair, or 3s. 3d. the half-dozen. They are made specially for hospital use, and are called the Victoria.

Uniform.—*Probationer* asks: Can you tell me where I can obtain information as to the style, colour, &c., of indoor and outdoor uniform worn at Kimberley Hospital, South Africa?—The nurses and probationers of the Kimberley Hospital wear black stuff dresses, white caps, aprons, collars, and cuffs. The outdoor uniform is black, the cloak being of thin material in summer and thicker in winter. A veil is worn with the bonnet.

### Everybody's Opinion.

### SO-CALLED NURSES' CO-OPERATION SOCIETIES.

"Interested" writes: I should esteem it a great favour if you would inform me through the correspondence columns of The Hospital how the conditions (terms) stated below, which are in force at an institute where the nurses receive their own fees, compare with the condition in force at other similar institutions in London. I may say that the charge of 5s. per week when out of a case and non-resident seems unfair, since the guinea per annum membership fee should cover and insure a nurse's name being kept on the roll. Nurses supply their own uniform. Conditions: 21s. per annum membership; 25 per cent. of fees received for first three months; 12½ per cent. of fees received after first three months; 15s. per week when out of a case and resident at the institute; and 5s. per week when out of a case and not resident at the institute.

[It is not evident why the word "Co-operation" is applied to such a system as this. There is neither entrance nor membership fee charged by The Nurses' Co-operation, 8, New Cavendish Street, London. Fifteen shillings a week for board and residence between cases is a reasonable charge, but the demand for five shillings a week from non-residents is unusual. At institutions where nurses take their own fees, a percentage is charged for working expenses. At the Nurses' Co-operation this is 7½ per cent., but when only a comparatively small number of nurses are associated the percentage is necessarily higher.—Ed. *T.H.*]

### THE ROYAL NATIONAL PENSION FUND: WHAT NURSES THINK OF IT AND FIND IT TO BE.

Mrs. J. Garrett, The Home, Knowbury, Ludlow, Salop, writes: As some contributors to the sick pay allowance of the National Pension Fund for Nurses may wonder if it be a well-worked scheme, I should like to tell them my personal experience of the same. Having fractured my leg I applied at once for sick pay, and I can say that nothing could be better planned. On application the money always came by return of post. Some difficulties arose from living so far from my doctor, but these were all considered in the most courteous manner, and week by week I received messages of sympathy. I used to think that there might be a great deal of bother to get the sick pay; others may imagine the same. I thank you very much for having planned such a scheme. It would have been a serious thing for me without it, as I shall have a heavy doctor's bill, and have had to hire a donkey carriage to get about. If you think, sir, my acknowledgment of and gratitude for the kindness I received from the R.N.P.F. would be useful, I shall be glad for you to publish my name.

### OVER THE HOSPITAL WALL.

We have received a letter from Mrs. J. H. Byese, *née* Bassett, on this subject, which is too long for publication, and enough has already been said by both parties in this matter.

**For Book World for Women and Nurses, and Reading to the Sick, see overleaf.**

### The Book World for Women and Nurses.

[We invite Correspondence, Criticism, Enquiries, and Notes on Books likely to interest Women and Nurses. Address, Editor, THE HOSPITAL (Nurses' Book World), 428, Strand, W.C.]

"DISINFECTION." By MARY TRUMAN and EDITH SYKES. (Published by the Roxburgh Press, 32, Charing Cross. Price 3d.)

The object with which this little pamphlet has been written is a most laudable one—to supply a small book "containing simple directions for disinfecting, together with a review of the chief disinfectants." We can only regret that though in some respects excellent, in others it should be so deficient. Its perusal gives an impression of "snippiness." It attempts to cover too much ground, and is not sufficiently detailed or emphatic on essential points. Thus, in the directions about disinfection, this book, which professes to be a guide to an ignorant public, merely directs that clothes are to be soaked in "a" disinfectant; "some" disinfectant is to be added to excreta; "a weak" one is to be used for this purpose, and "a strong" one for that. To add to the confusion a long list of disinfectants is given, and we are told that camphylene, Cooper's salts, Mackey's oxychlorogene, chromic acid, are "disinfectants in common use"; whilst Quibell's pinosol oleusaban, Whalley's disinfecting fluid and concentrated sanitary powder, cupralum, ferralum, chloralum, are all recommended. Surely the perplexed public will exclaim, "It is very interesting to learn there are so many disinfectants, but how to know what is best for each special purpose, and what strength is to be used? Surely there is one disinfectant, to the exclusion of others, that is best for sponging a patient, another for disinfecting his clothes, and another for drains; what am I to do amidst this *embarras de richesse*?" The authors, in fact, actually say that one disinfectant may be used one day, and another kind the next. We are strongly of opinion that the only way to teach intelligent disinfection to the uninitiated is to recommend only some three or four disinfectants, the efficiency of which has been thoroughly and scientifically tested. In these days of disinfection by steam at high pressure, it would surely be advisable to state clearly that this can be secured in all large towns, giving no trouble whatever, and not necessitating the unmaking of bedding. Also for fumigating rooms the old plan of burning sulphur is the only one recommended, and no mention is made of the more recent method of using the liquified sulphurous acid, which avoids all trouble, risk of accident, or of the sulphur not burning properly. We hope a future edition of this little work may be thoroughly revised and brought up to date, so that it may really prove as useful as it promises to be.

NOTES ON NURSING. By Nurse EVELYN HERBERT (printed by T. Dobb and Co., 229, Brownlow Hill, Liverpool).

THIS book is a small one of only 93 pages, but it contains an unusual amount of valuable information. It is intended mainly for trained nurses, as a book for reference says the authoress, but certainly its usefulness is not confined to nurses. Notes on the methods of giving baths as well as the requisite temperature of each kind, are given with fair detail. With reference to the hot mustard bath, however, we notice that the quantity of water is not mentioned to which "about one ounce of mustard" is to be added, and also the comparative advantages of mixing the latter with hot or cold water are omitted. The paragraphs on the prevention and treatment of bedsores are good, although the duty of calling the doctor's immediate attention to their first appearance is omitted, possibly because it is considered too obvious to need mention. The directions as to nutrient enemata are somewhat incomplete, as they include only one method of injection, viz. the ball syringe. The notes on burns and scalds are admirably concise and practical. The little book contains an excellent glossary and many hints specially useful to private nurses. Altogether we can heartily commend the small volume to our readers' notice, for its virtues are many and its blemishes few. Amongst the latter we rank the statement that many hospitals are too poor to provide ward sheets and mackintoshes for the beds. Their absence we should certainly consider evidence of bad management.

### For Reading to the Sick.

### PEACE.

**Motto.**

THE blessed shall hear no vain words, but only the word *Peace.—Koran.*

**Verses.**

Grant peace on earth, and after we have striven,
Peace in Thy Heaven. —*P. Pusey.*

O Lord, how happy should we be
If we could cast our care on Thee;
If we from self could rest,
And feel at heart that One above
In perfect wisdom, perfect love,
Is working for the best.

How far from this our daily life,
How oft disturbed by anxious strife,
By sudden wild alarms;
* * * *
Could we but kneel and cast our load,
E'en while we pray upon our God,
Then rise with lightened cheer;
Sure that the Father, Who is nigh
To still the famished raven's cry,
Will hear in that we fear.

We cannot trust Him as we should,
So chafes weak nature's restless mood
To cast its peace away; . . .
Leave all things to a Father's Will,
And taste, before Him lying still,
E'en in affliction, Peace.
—*Joseph Anstice.*

Grant us Thy peace throughout our earthly life,
Our balm in sorrow and our stay in strife;
Then when Thy voice shall bid our conflict cease,
Call us O Lord to Thine eternal peace!
—*Ellerton.*

**Reading.**

Peace is what all desire, but all do not care for the things that pertain unto true peace.—*Thos. à Kempis.*

Is not all the world God's family? Are not we His creatures? Are we not as clay in the hand of the potter? Do we not live upon His meat, and move by His strength, and do our work by His light? Are we anything but what we are from Him? And shall there be a mutiny among the flocks and herds, because their lord or their shepherd chooses their pastures, and suffers them not to wander into deserts and unknown ways? If we choose, we do it so foolishly that we cannot like it long, and most commonly not at all; but God, Who can do what He pleases, is wise to choose safely for us, affectionate to comply with our needs, and powerful to execute all His wise decrees. Here therefore is the wisdom of the contented man, to let God choose for him; for when we have given up our wills to Him, and stand in that station of the battle where our great General hath placed us, our spirits must needs rest while our conditions have for their security the power, the wisdom, and the charity of God.

Contentedness in all accidents brings great peace of spirit, and is the great and only instrument of temporal felicity.—*Jeremy Taylor.*

Our whole peace in this miserable life consisteth rather in humble sufferance, than in not feeling oppositions.

He that can best tell how to suffer, will best keep himself in peace. That man is conqueror of himself, and lord of the world, the friend of Christ, and the heir of heaven.—*Thomas à Kempis.*

Thou shalt keep him in perfect peace whose mind is stayed on Thee.—*Isaiah.*

The peace of God which passeth all understanding keep your hearts and minds.—*St. Paul.*

